# Supplementary material for: Hybrid SiO2/Si pillar-based optomechanical crystals for on-chip photonic integration
Source: Nanophotonics. 2025 Jul 28;14(17):2953–61. doi: 10.1515/nanoph-2025-0232 (PMC12397729; doi:10.1515/nanoph-2025-0232)
Supplement: Supplementary file 1 — Supplementary Material Details [file j_nanoph-2025-0232_suppl_001.pdf]

Hybrid SiO_2/Si Pillar-Based Optomechanical Crystals for On-Chip Photonic Integration (Supplementary Material)

Martin Poblet[a], Christian Vinther Bertelsen[b], David Alonso-Tomás[a], Rahul Singh[b], Elena López-Aymerich[c], Jens Goldschmidt[d], Katrin Schmitt[d], Maria Dimaki[b], Winnie E. Svendsen[b], Albert Romano-Rodríguez[a,*], Daniel Navarro-Urrios[a,*]

[a] MIND-IN2UB, Departament d'Enginyeria Electrónica i Biomédica, Facultat de Física, Universitat de Barcelona, Martí i Franquès 1, Barcelona 08028, Spain
[b] DTU Bioengineering, Danmarks Tekniske Universitet (DTU), 2800 Kgs. Lyngby, Denmark.
[c] DTU Nanolab, Oersteds Plads – Building 347, Danmarks Tekniske Universitet (DTU), 2800 Kgs. Lyngby, Denmark.
[d] Institut für Mikrosystemtechnik – IMTEK, Albert-Ludwigs-Universität Freiburg, Georges-Köhler-Allee 102, 79110 Freiburg, Germany
*e-mails: albert.romano@ub.edu, dnavarro@ub.edu

S1 Fabrication details

The one-dimensional photonic crystal (1D-PhC) pillar cavities were fabricated using electron beam lithography and reactive ion etching (RIE) on a silicon-on-insulator (SOI) wafer (see Figure S1). The wafer featured a 1.5 µm silicon device layer atop a 2 µm thick buried silicon oxide layer and was acquired from Siegert Wafer GmbH (Germany). The device layer comprised <100>crystalline p-type silicon with a specified resistivity of 1-5 Ohm-cm. The preparation for electron beam exposure involved spin coating a 180 nm layer of CSAR positive resist (AR-P 6200, Allresist GmbH, Germany), followed by thermal evaporation of a 20 nm aluminum discharge layer. The pattern was exposed using a JEOL JBX-9500FS electron beam lithography system at a dose of 350 µC/cm². The discharge layer was removed using the TMAH developer (AZ 726 MIF, Merck, Germany), and the pattern was developed with AR 600-546 (Allresist GmbH, Germany) for 90 seconds. Subsequently, a 40 nm aluminum layer was deposited by e-beam evaporation (Temescal Systems, Ferrotec). A lift-off process using Microposit Remover 1165 removed the CSAR resist, leaving the aluminum layer as a mask for dry etching. Quality checks of the aluminum mask, including pattern shape and uniformity, were performed using SEM. RIE was carried out with an SPTS-Pegasus etcher in a

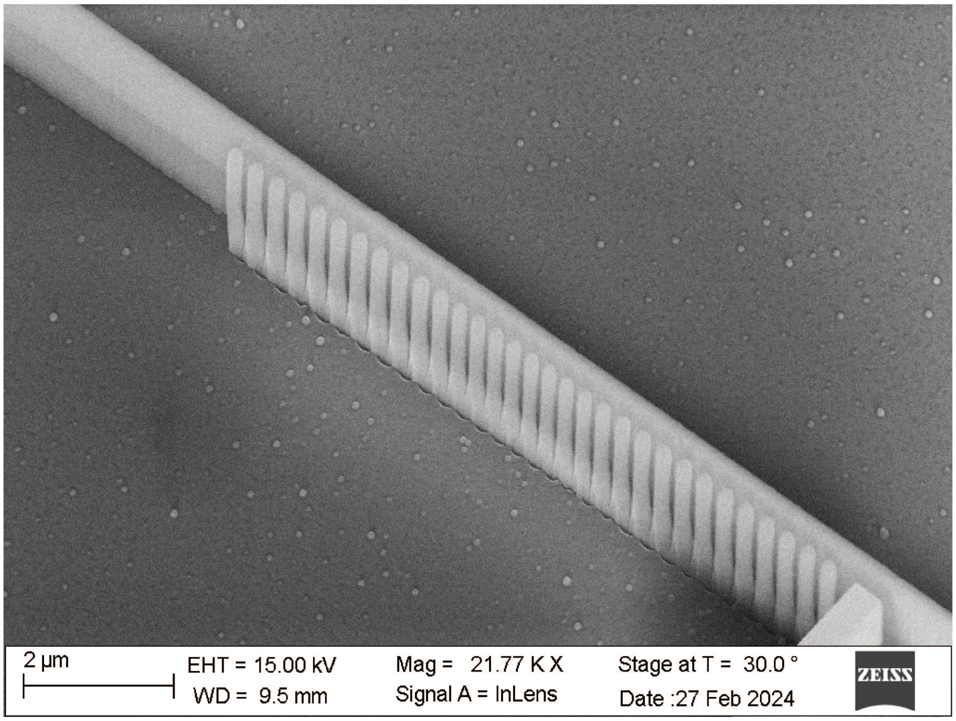

Fig. S1: Scanning electron microscopy image of one of the fabricated samples.

single processing step, utilizing a gas mixture of SF6 (at 44 sccm) and C4F8 (at 77 sccm). The etch process lasted 5-6 minutes with a coil power of 1000 W and a platen power of 20 W, until the buried oxide was reached and the silicon device layer was fully removed. The processing parameters were then adjusted (40 sccm C4F8, 5 sccm O2, coil power 1100 W, platen power 180 W) to etch the oxide for 2.5 minutes using an STS-MESC Multiplex ICP etcher, achieving an etch depth of approximately 1 µm. Finally, the remaining aluminum mask was removed with TMAH developer.

S2 Optimal coupling for enhanced transduction in direct detection

In the main text, we explained the methodology used to optimize the coupling distance between the waveguide and the cavity. Here, we present the derivation of the intra-cavity power expressions provided in the text.

We begin with the Langevin equation [1]:

$$\frac{da(t)}{dt} = \left(-i\Delta - \frac{\kappa}{2}\right) a(t) + \sqrt{\eta\kappa}\, a_{\text{in}}(t) \tag{S1}$$

where $\Delta = \omega - \omega_c$ is the detuning, κ is the total decay rate, η the coupling coefficient and $a_{\text{in}}(t)$ is the incident field amplitude. In the steady state:

$$a = \frac{\sqrt{\kappa\eta}}{\kappa/2 + i\Delta}\, a_{\text{in}} \tag{S2}$$

The intra-cavity power then reads:

$$P_{\text{cav}} = \hbar\omega|a|^2 = \hbar\omega \frac{\eta\kappa}{\Delta^2 + (\kappa/2)^2}|a_{\text{in}}|^2 \tag{S3}$$

On resonance ($\Delta = 0$) and for both, unidirectional ($\eta = \kappa_e/\kappa$) or bidirectional ($\eta = \kappa_e/2\kappa$) coupling, we find:

$$P_{\text{cav}} \propto \frac{\eta}{\kappa} \propto \frac{\kappa_e}{(\kappa_i + \kappa_e)^2} \tag{S4}$$

By including the expression for κ_e in Eq. S4, we arrive at the equation presented in the main text for the intra-cavity power. The value of d that maximizes this expression is d_c.

Now, we aim to perform a similar analysis, but focusing on optomechanical transduction rather than intra-cavity power. The mechanical displacement x shifts the cavity resonance frequency (ω_c). In a linear approximation, this is given by $\Delta = \Delta_0 - Gx$, where $G = \frac{d\omega_c}{dx}$ is the optomechanical coupling coefficient.

We are interested in how changes in x affect the output transmission which read as [2]:

$$T = 1 - \frac{\kappa^2\eta(1-\eta)}{(\Delta_0 - Gx)^2 + (\kappa/2)^2} \tag{S5}$$

Taking the derivative, we can define the transduction sensitivity in direct detection:

$$S \propto \left|\frac{dT}{dx}\right| = \frac{2G\kappa^2\eta(1-\eta)|\Delta|}{(\Delta^2 + (\kappa/2)^2)^2} \tag{S6}$$

The problem now reduces to finding the maximum of this function deppending on detuning, which occurs at $|\Delta| = \kappa/(2\sqrt{3})$. At this point, the maximum sensitivity scales as:

$$S_{max} \propto G\frac{\eta(1-\eta)}{\kappa} \tag{S7}$$

Interestingly, here the dependence on κ_e is influenced by the type of coupling.

Unidirectional coupling ($\eta = \kappa_e/\kappa$)

Under this scheme, the maximum optomechanical transduction in direct detection read as:

$$S_{max,u} \propto \frac{\kappa_e}{(\kappa_e + \kappa_i)^3} \tag{S8}$$

Note the difference in scaling compared to the intra-cavity power: in this case, the denominator scales cubically, whereas for the power it was quadratic. The factor

$F(\kappa_e) = \kappa_e/(\kappa_e + \kappa_i)^3$ reaches its peak at $\kappa_e = \kappa_i/2$, in contrast to the critical coupling condition for maximum intra-cavity power. Substituting the expression for κ_e, the corresponding coupling distance that yields this optimal condition is:

$$d_{OM,u} = d_c + d_0 \ln 2 \tag{S9}$$

Bidirectional coupling ($\eta = \kappa_e/2\kappa$)

In this scenario, typical of one-dimensional photonic crystal cavities [2], the maximum sensitivity scales as:

$$S_{max,b} \propto \frac{\kappa_e(\kappa_e + 2\kappa_i)}{(\kappa_e + \kappa_i)^3} \tag{S10}$$

which maximizes when $\kappa_e = (\sqrt{3} - 1)\kappa_i \approx 0.73\kappa_i$, resulting in an optimized coupling distance of:

$$d_{OM,b} = d_c - d_0 \ln(\sqrt{3} - 1) \tag{S11}$$

S3 Ideal cavity simulations

To illustrate the dependence of the intrinsic quality factor on d_c, we repeat the analysis performed in the experiment using a simulated cavity with additional losses removed. Figure S2 shows the result of this analysis, where the squared ratio between the intra-cavity (E_{cav}) and waveguide (E_{wav}) electric fields is plotted as a function of the incident wavelength for different coupling distances (Fig. S2a). The quality factor and resonance amplitude are extracted using a Lorentzian fit and are plotted in Figs. S2b and S2c, respectively.

First, we note that in the absence of induced absorption, the intrinsic quality factor is one order of magnitude higher than that observed in the experiment. We fit both quantities using the expressions derived in the main text, assuming an evanescent dependence of κ_e on distance. Notably, the critical distance obtained from fitting the quality factor data does not coincide with the distance yielding maximum intra-cavity power (see Fig. S2). We attribute this deviation between the theoretical model and the simulation data to boundary conditions or reflections that may introduce discrepancies when the waveguide is positioned at distances comparable to the size of the simulation domain.

In any case, we observe that the critical distance lies near 400 nm, significantly farther than the 250 nm obtained when including absorption to match the experimental quality factors.

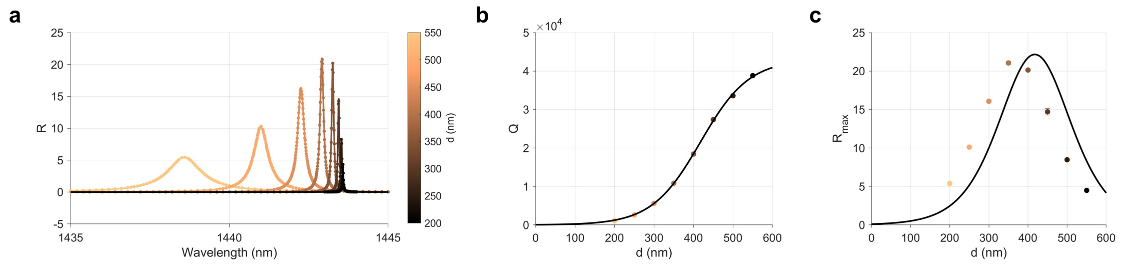

Fig. S2: Coupling distance simulations without considering additional losses. a) Ratio between E^2_{cav} and E^2_{wav}, i.e., R, for several gaps. b) Optical quality factor for several gaps. c) Maximum values of R for the curves of panel a.

S4. Influence of the Geometric Scaling Factor on the Optical Band Structure

To illustrate the influence of the geometric scaling factor **g** on the optical response of the cavity, we examine the photonic band structure of a single pillar unit cell designed for transverse magnetic (TM)-like polarization. The main panel of Figure S3 shows the calculated photonic band diagram, which corresponds to the band structure presented in Figure 1a of the main text.

In the right panel of Figure S3, we plot the energy of the optical modes at the Brillouin zone edge (X-point, $\kappa_x = \pi/a$) as a function of the scaling factor **g**. This parameter rescales both the pillar radius **r** and the lattice constant **a** uniformly, while keeping the pillar height fixed. As expected, reducing **g** shifts the photonic band edge to higher energies. This shift provides a straightforward mechanism for engineering a defect state within the photonic bandgap: by gradually tapering the pillar dimensions toward the center of the structure, a localized optical mode can be created.

Once known the band dispersion dependence with the geometrical parameters, to create the 1D-PhC cavity we follow the common procedure discussed elsewhere (see for example [3]), which leads to the 1D-PhC cavity geometry described in the main text.

S5. Influence of Multimode Interference on Optical Transmission and Mechanical Signal Detection

Figure S4 provides a side-by-side comparison of the transduced mechanical signal (panel a) and the optical transmission spectrum (panel b) as functions of the laser excitation wavelength. As described in the main text, the transmitted optical signal results from the interference between several optical modes supported by the waveguide structure—at least three in the narrower waveguide region (see also Figure 2a). This multimode interference gives rise to pronounced intensity oscillations in the transmission spectrum, with modulation depths exceeding 50% of the average signal. These oscillations obscure the cavity resonance, making it difficult to unambiguously identify its spectral position

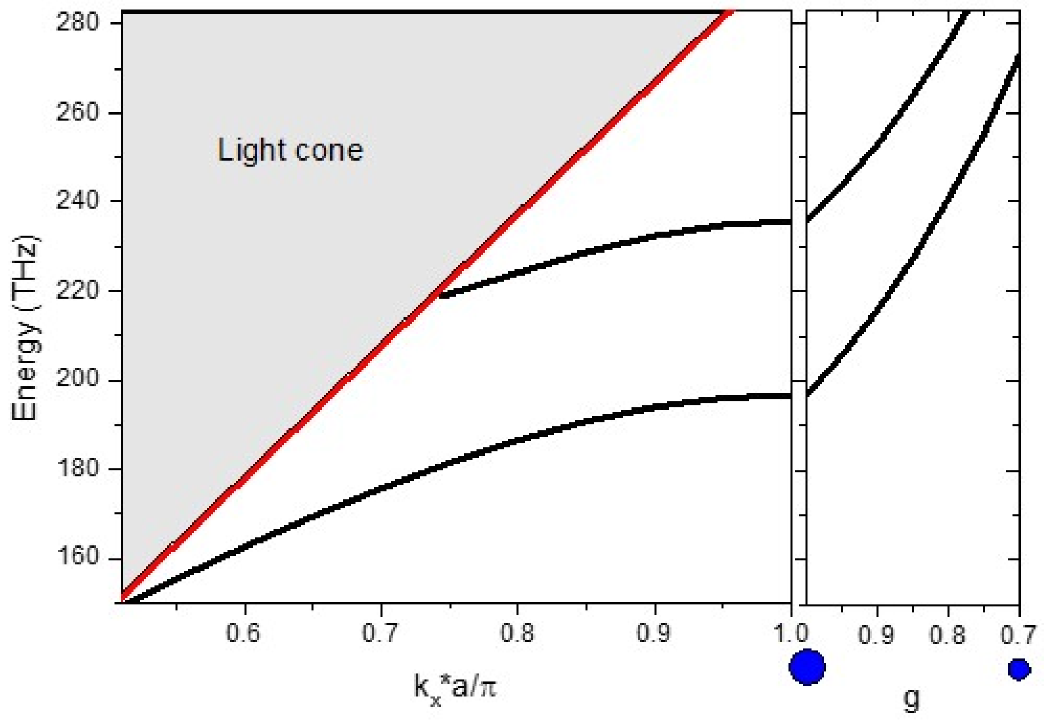

Fig. S3: Band diagram of the nominal pillar unit-cell. The right side panel illustrates the dependence of the X-point band edge energy on the reduction factor g.

using transmission data alone.

It is important to emphasize that the optical coupling between the waveguide and the cavity is primarily optimized for the fundamental mode. Coupling to higher-order waveguide modes is highly inefficient, owing to the mismatch in the spatial field profiles, particularly along the axis parallel to the pillars. As a consequence, higher-order modes largely bypass the cavity and propagate through the waveguide without interacting significantly with it. Only the fundamental mode contributes effectively to the excitation of the cavity resonance.

In contrast to the optical transmission signal, the mechanical modes of the pillars forming the 1D-PhC cavity are clearly observable in the radio-frequency (RF) modulation spectrum. This is evident in the distinct RF peaks around 30 MHz shown in Fig. S4a. These mechanical oscillations are detected only when the laser wavelength is resonant with the optical cavity, allowing for precise determination of the resonance wavelength and linewidth—1363.6 nm and approximately 0.5 nm, respectively, in the example shown.

While the transmission spectrum may offer insight into coupling efficiency and optical quality factors, the key information for our sensing scheme is already encoded in the RF response. Within the scope of our approach—where the transduction of

mechanical motion serves as the primary sensing mechanism—interference features from multimode propagation act as passive optical filters. In particular, under destructive interference conditions, these features suppress the optical component of the signal that couples to the mechanical modes, reducing the efficiency of transduction.

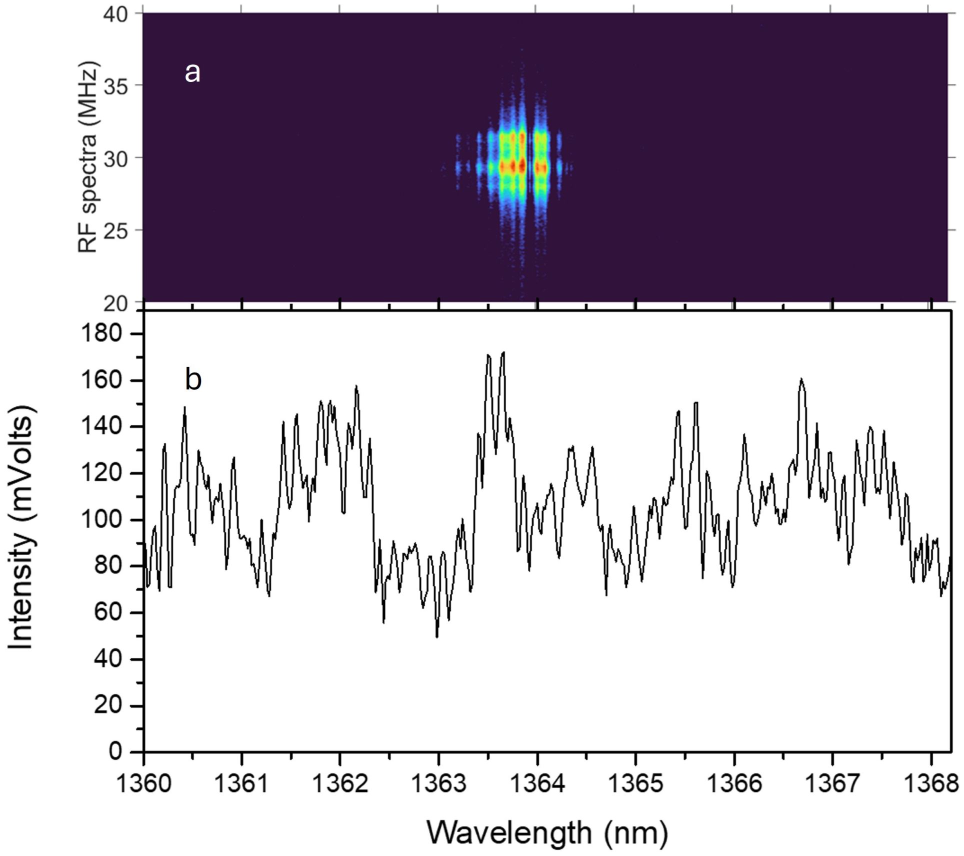

Fig. S4: Comparison between the excitation wavelength dependence of the transduced RF signal (panel a) and the transmission signal (panel b). Both panels share the same horizontal axis..

S6 Simulation Procedure

Here we show the procedure used to compute each of the wavelength sweeps using a self-adjusted step size. A flowchart of the entire procedure is shown in Fig. S5. First, we define the sweep parameters, including the initial and final wavelengths, the gap, and the initial step size. For each selected wavelength, the ratio R (as defined earlier) is computed in COMSOL Multiphysics using the Simulink interface between MATLAB and COMSOL. The outputs are stored in a file.

Next, the data is iterated and the differences between consecutive values of R, i.e., $R_{i+1} - R_i$, are compared against a predefined absolute tolerance. As long as this

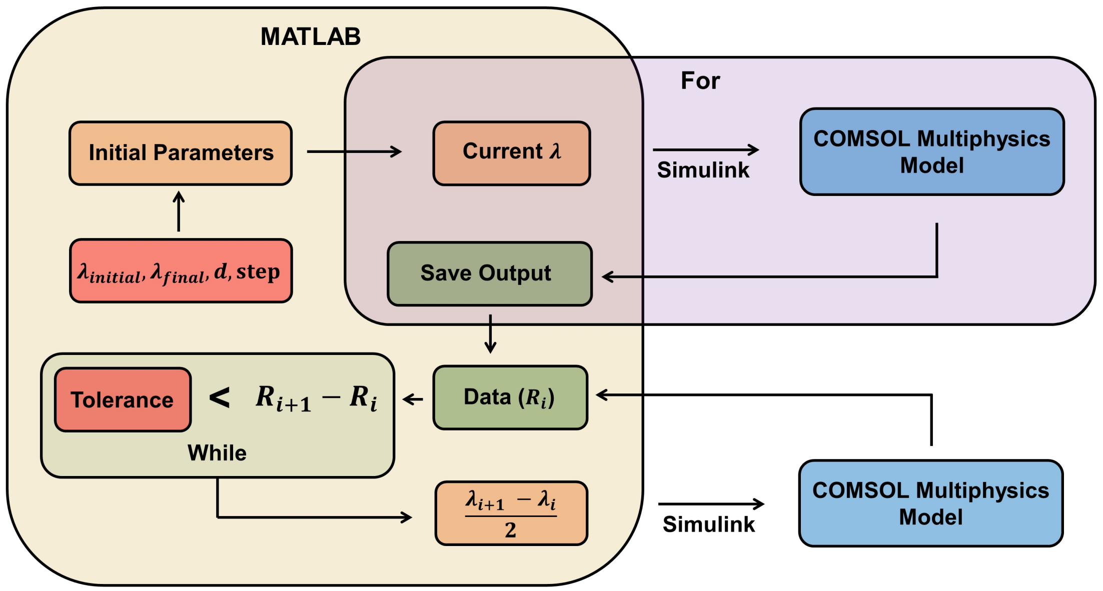

Fig. S5: Flowchart of the simulation.

variation exceeds the threshold, additional values of R are computed at intermediate wavelengths between each pair λ_{i+1} and λ_i.

This approach allows us to automate multiple sweeps for each distance without requiring an extremely small initial step size or an initial search for the location of the optical mode.

S7 Estimation of the responsivity of the mechanical eigenfrequencies to applied force derivatives.

To quantitatively estimate the response of mechanical frequencies to force gradients induced by deformation, we performed FEM simulations. An elastic boundary condition was applied to the top surface of a pillar (see right panel of Fig. S6), and the mechanical resonance frequency was tracked as a function of the applied force derivative (main panel of Fig. S6). These simulations reveal that the fundamental mode family is approximately 10 times more compliant to force derivatives than the second-order modes. This difference arises from the distinct deformation profiles associated with each mode type. Specifically, the responsivities of the fundamental and second-order modes are about 10 [MHz·m/N] and 1 [MHz·m/N], respectively. Although the fundamental modes have lower mechanical quality factors compared to higher-order modes, their greater responsivity results in improved sensitivity—that is, a lower minimum detectable force gradient. Sensitivity, defined as the force derivative required to produce a frequency shift larger than the resonance linewidth, is higher for the fundamental modes, with detection thresholds below 50 mN/m.

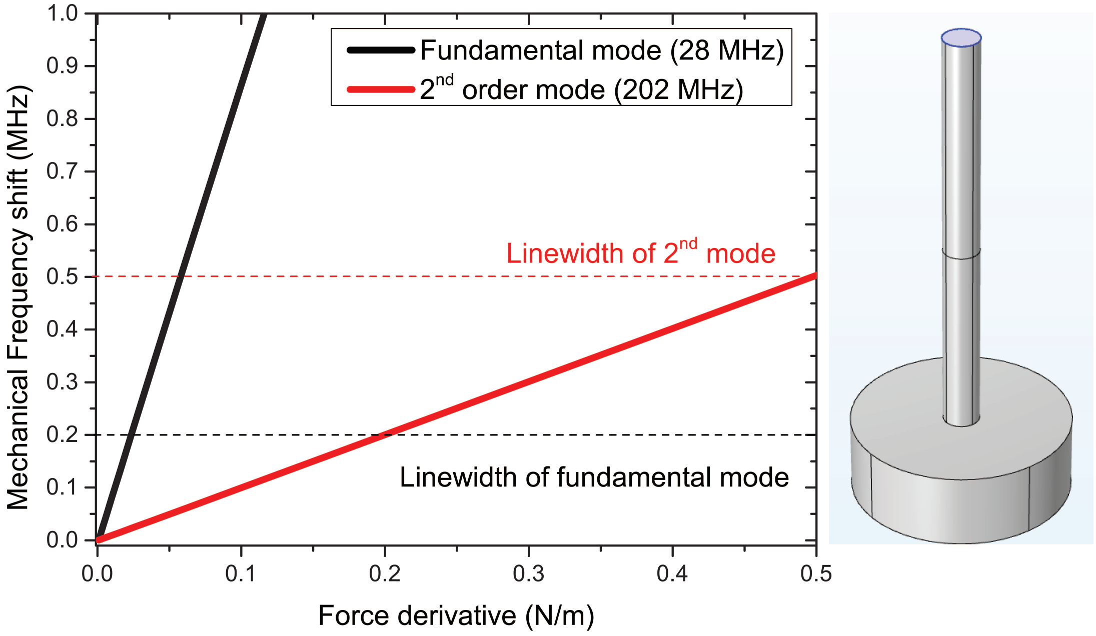

Fig. S6: Finite element method simulations of the response of the mechanical eigenfrequency of a nanopillar to force derivatives applied on the top part of the pillar . The black and red curves correspond to the first and second order mode, respectively. Horizontal dashed lines correspond to the experimental mechanical linewidths of the mechanical modes. The right panel sketches the geometrical FEM model, with the blue surface indicating the spring foundation node.

References

[1] Aspelmeyer M, Kippenberg T and Marquardt F. (2014). Cavity optomechanics. *Rev.Mod.Phys.* **86**(4), 54

[2] Hill, Jeffrey Thomas (2013) Nonlinear Optics and Wavelength Translation Via Cavity-Optomechanics. Dissertation (Ph.D.), California Institute of Technology. doi:10.7907/DKW6-TF64.

[3] Eichenfield, M., Chan, J., Camacho, R. et al. Optomechanical crystals . Nature 462, 78–82 (2009).
